# Handbuch der Zahnheilkunde, etc.

**Published:** 1842-12

**Authors:** 


### Bibliographical Notices.

*Handbuch der Zahnheilkunde, etc. etc. etc.* Von C. J. Linderer, Sé , Konigl. Preuss. approb. zahnarzte, Furstlich waldeckschen Hofzahnarzte, Universitats zahnarzte zu Gottingen, etc. etc. etc., und Joseph Linderer, Konigl. Preuss. approb. zahnarzte, etc. etc., zweite vermhrte und veranderte Austage Von J. Linderer. Mit achtzehn Lithographirten Tabellen. Berlin, 1842, 8vo. pp. 502.

Although the Prussians have not contributed as largely as the French, English, or even Americans to the literature of dental surgery, we are nevertheless indebted to them for some very valuable works. As long ago as 1804, a work of 564 8vo pages, written by J. J. J. Serre, was published in Berlin, and although in point of merit, nearly, if not quite equal to any treatise that had at that time appeared on practical dentistry, it has been passed by in silence, by almost every subsequent writer.

With regard to the work now before us, we have not as yet been able to read the whole of it, but from the portions of it which we have perused, we believe it to be one of considerable merit. The authors are certainly men of some science, and the fact that the work has so soon passed into a second edition, the first having been published in 1837, is at least an evidence that it is well thought of in its own country.

The greatest fault which we find with it is, that it is too brief. It does not treat upon the subjects embraced in it, at sufficient length. But taking it as a whole, we are disposed to believe it an excellent "Manual of Dental Surgery," and would be highly gratified to see it in an English dress.

It treats upon the various subjects connected with Dental Anatomy, Physiology, Materia Medica, and Surgery. The plates that accompany it are lithographic, and executed in a very superior manner.

